# Etiology and Epidemiology of Diarrhea in Hospitalized Children from Low Income Country: A Matched Case-Control Study in Central African Republic

**DOI:** 10.1371/journal.pntd.0004283

**Published:** 2016-01-05

**Authors:** Sébastien Breurec, Noémie Vanel, Petulla Bata, Loïc Chartier, Alain Farra, Loïc Favennec, Thierry Franck, Tamara Giles-Vernick, Jean-Chrysostome Gody, Liem Binh Luong Nguyen, Manuella Onambélé, Clotaire Rafaï, Romy Razakandrainibe, Laura Tondeur, Vianney Tricou, Philippe Sansonetti, Muriel Vray

**Affiliations:** 1 Laboratoire de Bactériologie médicale, Institut Pasteur de Bangui, Bangui, République Centrafricaine; 2 Laboratoire de Microbiologie Clinique et Environnementale, Centre Hospitalo-Universitaire, Pointe-à-Pitre/Les Abymes, Guadeloupe, France; 3 Université des Antilles, Faculté de Médecine, Pointe-à-Pitre, Guadeloupe, France; 4 Unité d’épidémiologie et d’expertise des maladies émergentes, Institut Pasteur, Paris, France; 5 Complexe Pédiatrique de Bangui, Bangui, République Centrafricaine; 6 Faculté de Médecine et de Pharmacie, Université de Rouen, Rouen, France; 7 Laboratoire de Virologie, Institut Pasteur de Bangui, Bangui, République Centrafricaine; 8 Unité de pathogénie microbienne moléculaire, Institut Pasteur, Paris, France; Massachusetts General Hospital, UNITED STATES

## Abstract

**Background:**

In Sub-Saharan Africa, infectious diarrhea is a major cause of morbidity and mortality. A case-control study was conducted to identify the etiology of diarrhea and to describe its main epidemiologic risk factors among hospitalized children under five years old in Bangui, Central African Republic.

**Methods:**

All consecutive children under five years old hospitalized for diarrhea in the Pediatric Complex of Bangui for whom a parent’s written consent was provided were included. Controls matched by age, sex and neighborhood of residence of each case were included. For both cases and controls, demographic, socio-economic and anthropometric data were recorded. Stool samples were collected to identify enteropathogens at enrollment. Clinical examination data and blood samples were collected only for cases.

**Results:**

A total of 333 cases and 333 controls was recruited between December 2011 and November 2013. The mean age of cases was 12.9 months, and 56% were male. The mean delay between the onset of first symptoms and hospital admission was 3.7 days. Blood was detected in 5% of stool samples from cases. Cases were significantly more severely or moderately malnourished than controls. One of the sought-for pathogens was identified in 78% and 40% of cases and controls, respectively. Most attributable cases of hospitalized diarrhea were due to rotavirus, with an attributable fraction of 39%. Four other pathogens were associated with hospitalized diarrhea: Shigella/EIEC, Cryptosporidium parvum/hominis, astrovirus and norovirus with attributable fraction of 9%, 10%, 7% and 7% respectively. Giardia intestinalis was found in more controls than cases, with a protective fraction of 6%.

**Conclusions:**

Rotavirus, norovirus, astrovirus, Shigella/EIEC, Cryptosporidium parvum/hominis were found to be positively associated with severe diarrhea: while Giardia intestinalis was found negatively associated. Most attributable episodes of severe diarrhea were associated with rotavirus, highlighting the urgent need to introduce the rotavirus vaccine within the CAR’s Expanded Program on Immunization. The development of new medicines, vaccines and rapid diagnostic tests that can be conducted at the bedside should be high priority for low-resource countries.

## Author Summary

Infectious diarrhea is a major cause of illness and death among children under five years from low-income country. In order to identify infectious agents associated with diarrhea, we conducted a case-control study in the Pediatric Complex of Bangui, the sole public pediatric hospital from Central African Republic (CAR). A total of 333 hospitalized children with diarrhea and 333 controls were included, controls being pair matched to the cases according to age, sex and neighborhood. At least one of the sought-for pathogens was identified in 80% of hospitalized children, and approximately one in ten cases presented mixed bacterial/viral co-infections. Five pathogens were positively associated with hospitalized diarrhea, namely rotavirus, norovirus, astrovirus, *Shigella*/EIEC and *Cryptosporidium hominis/parvum*. *Giardia intestinalis* was negatively associated with hospitalized diarrhea. A seasonality effect—viruses during the dry season, bacteria and parasites during the rainy season—but also an age effect, were observed, which may guide clinicians in the management of diarrhea. As rotavirus was the leading cause of severe diarrhea, the introduction of the rotavirus vaccine in CAR will certainly provide considerable direct health benefits in terms of reduced illness and deaths. New medicines, vaccines and rapid diagnostic tests that can be conducted bedside should be urgently developed for low-resource countries.

## Introduction

In 2013, 6.3 million children under the age of five years died, 578,000 of them from diarrheal diseases. Nearly half of these diarrhea-related deaths were reported from Sub-Saharan Africa [1]. The fourth Millennium Development Goal, established after the United Nations Millennium Summit in 2000, seeks to decrease the mortality of children under five by two-thirds before 2015 [2]. Since 2000, childhood mortality due to diarrhea has diminished by 6.5% every year, but this trend requires an acceleration to reach the 2030 objectives. In order to achieve this decline in childhood diarrheal mortality, the World Health Organization (WHO) published guidelines for the clinical management of childhood diarrhea [3]. These guidelines recommend using antibiotics only for bloody diarrhea, suspected cholera, or associated sepsis. They also encourage zinc supplementation and use of oral rehydration solution (ORS) to treat and prevent diarrhea. However, in practice, antibiotic treatments are overused, resulting in the emergence of antibiotic resistance: only 40% of the children with diarrhea receive the recommended zinc supplementation and ORS [4].

Nowhere are these problems of childhood diarrhea and shortfalls in its management more evident than in the poorest and most unstable countries of Sub-Saharan Africa. The Central African Republic (CAR) is a resource-limited country in equatorial Africa (ranked 180/187 according to the Human Development Index in 2013). Mortality among under five year-old children was 179/1000 in 2010 [5]. No high-quality epidemiological and biological data on severe childhood diarrhea, however, exist for CAR. Indeed, the consequences of decades of poverty, civil war, and economic and political crisis have complicated the management of severe childhood diarrhea. Moreover, a recent qualitative investigation in CAR revealed the complex home management of childhood diarrhea. Parents’ beliefs that diarrheal illness needs to be stopped immediately, that it requires medication, that they should avoid consulting primary health centers and minimize expenses were the most important reasons hampering effective home management of diarrhea [6]. The CAR therefore provides a strong case study in which to understand better the epidemiology and etiology of severe childhood diarrhea in the poorest and most unstable countries without functioning health care systems. Investigating childhood diarrhea in such a context can assist in managing this treatable pathology, by highlighting the most appropriate and adaptive public health interventions.

The Global Enteric Multicenter Study (GEMS) study is a matched case-control study of moderate-to-severe diarrhea in children aged 0–59 months which aimed to estimate the pathogen-specific disease burden in populations from four sites in Africa and three in Asia. The GEMS showed that preventive strategies targeting five pathogens (rotavirus, *Shigella*, ST-ETEC, *Cryptosporidium*, typical enteropathogenic *E coli*) are likely to substantially reduce the burden of moderate-to-severe diarrhea. However, the public health interventions in very low income countries like the CAR, suffering from long-term instability, are different from relatively stable countries with higher income and better medical infrastructure.

Our study, a matched case-control study of diarrhea among hospitalized children under five years was conducted at the Pediatric Complex (PCB) in the CAR’s capital city, Bangui. It was performed in collaboration with the Institut Pasteur de Paris (IPP), the Institut Pasteur de Bangui (IPB) and the PCB.

The study’s primary objective was to identify pathogens associated with diarrhea in hospitalized children under five years of age. Secondary objectives were i) to describe the clinical symptoms of severe diarrhea among hospitalized children, ii) to identify the risk factors associated with severe diarrhea (anthropometric, socio-economic, environmental characteristics), iii) to describe the management of diarrhea before and during hospitalization, and iv) to describe the vital status of children with severe diarrhea during hospitalization and two months after discharge.

## Methods

### Study design and setting

Our study was a matched case-control study conducted in Bangui, CAR from December 2011 to November 2013 at the PCB, the country’s sole public pediatric hospital. Cases were children under 60 months of age, hospitalized for diarrhea. Other inclusion criteria were residence in one of Bangui’s eight districts and a general health condition that would support blood and stool sampling. Exclusion criterion was being positive for human immunodeficiency virus (HIV). Controls, identified from the community, were pair matched to the cases according to age (±2 months for infants (0–11 months), ±3 months for toddlers (12–23 months) and ±6 months for children (24–59 months)), sex and neighborhood. To be eligible, controls had to be in good general health, with no history of diarrhea or antibiotic use during the seven days before sampling. HIV status was not systematically tested in controls, but if parents spontaneously declared a child to be seropositive, that child was not included. Cases and controls could not be included more than once.

### Ethics

The research protocol was approved by the Scientist Committee of the Sciences and Health University of Bangui, the CoRC (Clinical Research Committee of Institut Pasteur), the CCTIRS (Comité Consultatif sur le Traitement de l’Information en matière de Recherche dans le domaine de la santé) and the CNIL (Commission Nationale de l’Information et des Libertés) in France. Written informed consent was obtained from all children’s parents or legal guardians for both cases and controls. This study was conducted according to the protocol and ethical principles with their origins in the Declaration of Helsinki. The project provided treatment and laboratory testing free of charge. The CN/CNLS (Coordination Nationale du Comité National de Lutte contre le Sida), with financial support from the Global Fund, covered all costs for HIV treatment.

### Data collection

#### Clinical cases

A pediatrician from the CPB carried out the clinical examination of potential cases and decided on the need for hospitalizing each case for severe diarrhea. Clinical observations, including signs of severe dehydration, hemodynamic shock and extra-digestive symptoms, were collected. At the inclusion of each case, a trained nurse conducted a standardized questionnaire with parents or caregivers so as to gather demographic, environmental and clinical information. Anthropometric measures were also collected: weight, length or height and mid-upper arm circumference (MUAC). Treatments used before and during hospitalization were recorded, as well as the duration of hospitalization and status at discharge. Two months after the child was discharged, parents were contacted by phone so that we could assess the child’s vital status.

#### Controls

At the inclusion of each case, the same trained nurse conducting the standardized questionnaire for all cases was asked to select age- and gender-matched children without diarrhea over the previous seven days, living in the same urban neighborhood as the case. Two nurses were responsible for control enrollments. Parents or primary caretakers underwent the same standardized questionnaire to collect demographic and epidemiological data. The same anthropometric measures were also gathered. Each control provided a fresh stool sample at home in the morning. The sample was returned within 30 minutes of collection to the Institut Pasteur laboratory. Mayors and chiefs of Bangui’s urban districts were informed of the implementation of the study; in turn, they informed the populations living in these districts. Among controls, the vital status at two months was not collected.

### Laboratory testing

#### Bacteriological diagnostic tests

Bacterial agents (*Salmonella enterica*, *Shigella* spp., *Campylobacter* spp., *Escherichia coli*, *Aeromonas* spp., *Vibrio cholerae*, *Yersinia enterocolitica*) were detected, as previously described [7]. Briefly, stool specimens were inoculated on Hektoen Enteric agar (Bio-Rad, Marnes-la-Coquette, France) to isolate *Shigella* species and *Salmonella enterica*, and on bromocresol purple lactose agar and Levine's eosin-methylene blue agar (Bio-Rad, Marnes-la-Coquette, France) to isolate *Escherichia coli* and other Gram-negative bacteria. Preston broth medium was inoculated to enrich *Campylobacter* species and subcultured into Karmali selective agar (Oxoid, Basingstoke, England). *Campylobacter* spp. detection was performed on around 25% of the stools due to reagent shortage. Muller—Kauffmann tetrathionate broth (Bio-Rad, Marnes-la-Coquette, France) was used to enrich *Salmonella* and subcultured into Hektoen Enteric agar. Serogrouping and serotyping of *Shigella* isolates were conducted with commercial antisera (Statens Serum Institut, Copenhagen, Denmark and Denka-Seiken, Tokyo, Japan, respectively). *Salmonella* isolates were serotyped according to the Kauffmann—White scheme using commercial antisera (Statens Serum Institut, Copenhagen, Denmark). Cefsulodin-irgasan-novobiocin agar (Bio-Rad, Marnes-la-Coquette, France) was used for *Yersinia enterocolitica* detection, and thiosulfate—citrate—bile salts—sucrose agar (Bio-Rad, Marnes-la-Coquette, France) for detection of *Vibrio cholerae* after selective enrichment with alkaline peptone water.

Five putative *E*. *coli* colonies from every stool were pooled and analyzed by a single-test multiplex PCR to identify intestinal pathotypes of *E*. *coli*, as previously described [8]. These pathotypes include enteropathogenic *E*. *coli* (EPEC), atypical EPEC (ATEC), Shiga toxin-producing *E*. *coli* (STEC), enterotoxigenic *E*. *coli* (ETEC), entero-invasive *E*. *coli* (EIEC) and entero-aggregative *E*. *coli* (EAEC). The following gene targets defined each E. coli pathotype: typical EPEC (*bfpB* positive), atypical EPEC (*escV* positive, *bfp* negative, *stx* negative), STEC *(escV* positive/negative, *bfp* negative, *stx1* positive, *stx2* positive, or both), ETEC (*elt* positive, *estIa* positive, *estIb* positive), EIEC (*invE* positive) and EAEC (*aggR* positive or *astA* positive with pic positive). Simultaneous detection of the *E*. *coli*-specific *uidA* gene was used to confirm biochemical identification of the isolate as *E*. *coli*, and to serve as PCR control.

DNA from faecal samples was extracted with the QIAamp DNA Stool Mini Kit (Qiagen, Courtaboeuf, France). To improve assessment of the involvement of *Shigella*, we performed a PCR assay based on amplifying the invasion plasmid antigen H (*ipaH*) gene contained in the four *Shigella* species and in EIEC [9].

#### Viral diagnostic tests

The stool specimens obtained from enrolled children were later tested for rotavirus, astrovirus, adenovirus and norovirus antigens by using commercial enzyme immunoassays. Suspensions of the stool specimens were prepared according to the manufacturer’s recommendations. For the detection of rotaviruses of the group A, astroviruses and adenoviruses, the ProSpecT Rotavirus, Astrovirus and Adenovirus tests (Oxoid, Thermo Fisher Scientific, Basingstoke, UK) were respectively used according to the manufacturer’s instructions. To detect noroviruses of the genogroups (GG) I and II, the IDEIA Norovirus kit (Oxoid, Thermo Fisher Scientific, Basingstoke, UK) was used according to the manufacturer’s instructions.

#### Parasite diagnostic tests

A portion of stool was concentrated by the merthiolate iodine formaldehyde concentration technique and examined for helminth eggs and protozoa cysts. Differentiation of pathogenic *Entamoeba histolytica* from non-pathogenic *Entamoeba dispar* was performed by enzyme-linked immunosorbent assay (ELISA) (Fumouze Diagnostics, France). Multiplex PCR was used to detect both *Cryptosporidium hominis* and *Cryptosporidium parvum* on extracted DNA from feces. Restriction enzyme digestion of PCR products to distinguish the two species of *Cryptosporidium* was not performed [10].

#### Blood tests

A blood sample of 10 mL maximum was collected from each case and sent at room temperature to the IPB within one hour. Blood smears were prepared, stained with 4% Giemsa and analyzed under a light microscope (×100 oil immersion) to detect forms of *Plasmodium* species. Hemogram using a Horiba ABX Pentra 80 and measure of the C-reactive-protein using an ABX Pentra 400 (Horiba ABX Diagnostics Inc., Montpellier, France) were realized for each patient. In addition, detection of HIV antibodies was performed using the Alere Determine^™^ HIV-1/2 Ag/Ab Combo (Orgenics, Israel) and the Vidas HIV Duo Quick (Biomerieux, France), in accordance with the manufacturer's instructions. Routine serologic testing is generally only informative before the age of 18 months if the test result is negative. Then, HIV-1 proviral DNA PCR (Biocentric, Bandol, France) was performed using the ABI PRISM 7000 real-time PCR system (Applied Biosystems, California, USA) in children younger than 18 months with positive HIV serologic test results. In children older than 18 months of age, HIV-1 and/or HIV-2 Western-blots (New Lav Blot I and II, Biorad, USA) were used to confirm seropositive anti-HIV-1 and/or anti-HIV-2 responses.

### Sample size calculation

To show an odds ratio of 2 characterizing the association between a given pathogen and severe diarrhea, with a pathogen prevalence of 5%, a power of 80% and a two-sided α = 0.05, a sample size of 600 cases and 600 controls was necessary.

### Statistical analyses

The variables collected are defined in Table 1. Data were double entered and analyzed using STATA: SE 13.1 (Stata Corp Station, TX, USA). Continuous variables were expressed as mean (±SD) or median [interquartile range] and discrete variable as percentage and 95% CI. Univariate analyses for continuous variables were performed using Student t-test or Mann-Whitney test when appropriate. For discrete variables, univariate analyses were performed using Chi-2 test or Fisher’s exact test. Tests were two-sided and a p-value<0.05 was considered significant.

**Table 1:** Definitions of the variables.

Variables	Definitions
Severe dehydration	One of the following signs: lethargy, unconsciousness or apathy; or two of the following signs: sunken and dry eyes, absence of tears, dry or sticky mouth, inability to drink, abdominal skin pinch with very slow recoil
Hemodynamic shock	One shock criteria according to age following the references of the francophone group of pediatric resuscitation [11]
Nutritional status^a^	Normal nutrition: MUAC ≥ 125mm^b^
	Moderate acute malnutrition (MAM): MUAC < 125mm and ≥ 115mm^b^
	Severe acute malnutrition (SAM): MUAC < 115 mm^b^
Weight for height Z-score (WHZ)	Calculated according to Myatt et al. [12]
Socio-economic level	Lowest income: no cell phone
	Middle income: cell phone but no car, refrigerator in working condition, or modern sanitation (flushing toilets inside the house)
	Highest income: car or refrigerator in working condition or modern sanitation (flushing toilets inside the house)
Seasons	Rainy season: from May to October
	Dry season: from November to April
Cohabiting parents	The child’s mother and father living in the same house
Improved water	Water from fountains or running water

^a^ The mid-upper arm circumference (MUAC) was chosen instead of the usual weight-for-height ratio for age because children with severe diarrhea can lose more than 10% of their body weight. In addition, the MUAC is less prone to errors, and thus a better indicator of mortality risk associated with malnutrition than weight-for-height [12].

^b^ According to WHO standards [13].

Comparison between cases and controls were made by univariate conditional logistic regression to take into account the matching of cases and controls. Pathogens potentially associated with severe diarrheas in univariate analysis with a p-value<0.25 were included in a backward conditional logistic regression, adjusted on the presence of other pathogens. Results are reported as adjusted OR (aOR) with 95% CI. The attributable fraction (AF) was calculated for pathogens with significant aOR with the following formula: aAF = P (pathogens) among cases* (aOR-1)/aOR, that is, the proportion of severe diarrhea attributable to this specific pathogen. When the association was significantly negative with an aOR<1, the protective fraction was calculated with the following formula: aPF = (1- aOR) x p (events) among controls, that is, the proportion of severe diarrhea avoided by the presence of this specific pathogen.

For cases, associations between socio-economic, anthropometric and clinical data with the use of ORS and antibiotics before and during hospitalization, and association with vital status were determined by univariate analysis. All variables associated with a p value<0.25 were included in a backward logistic regression. The final model includes only variables with a p-value<0.05. Interactions were tested and the goodness-of-fit of the model was studied using the Hosmer-Lemeshow statistic.

## Results

### Description of cases

**Fig 1:**
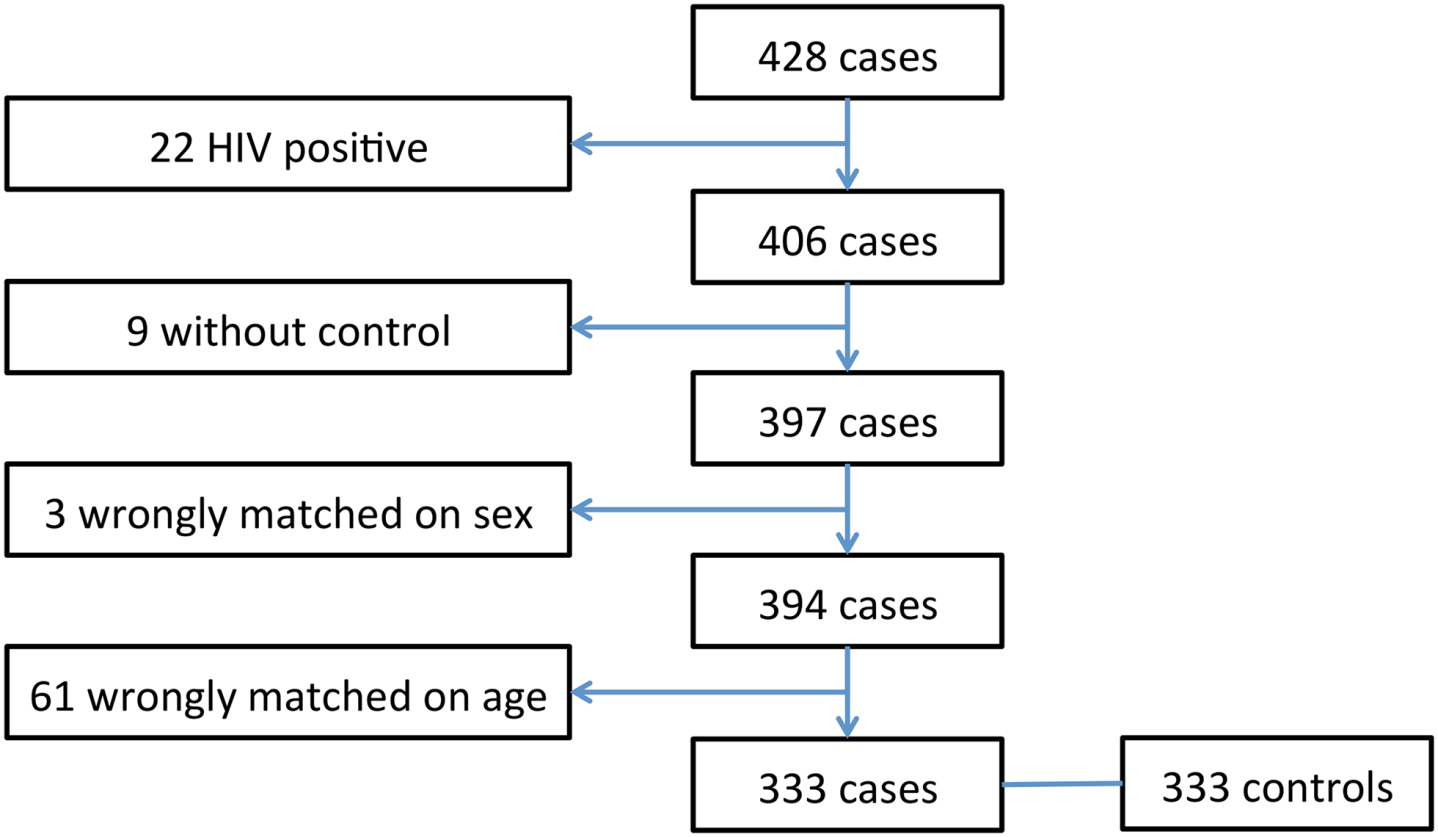
Study flow chart.

General characteristics of cases are described in Table 2. During the 24 months of the study, 428 consecutive cases were hospitalized for diarrhea. Twenty-two were tested positive for HIV and consequently excluded on that basis. No mothers refused their children’s participation in the study. Nine cases did not match any control. Sixty-four case-control pairs were wrongly matched: 3 for sex and 61 for age. Finally, 333 cases (78%) and 333 (79%) controls were analyzed (Fig 1). Fifty-six percent were male. The mean age at inclusion was 12.9 months (±9.8). The distribution of age was as follows: 195 (59%) between 0–11 months; 103 (31%) between 12–23 months and 35 (10%) between 24–59 months.

**Table 2:** Demographic characteristics of cases and controls (N/333 (%)).

*Characteristics*		*Cases (n = 333)*	*Controls (n = 333)*	*p-value*
*Male individuals*		*186 (56)*	*186 (56)*	
*Age in months*, *Mean (±SD)*		*12*.*9 (10)*	*14*.*1 (10)*	*0*.*15*
*Nutritional Status*	*SAM* ^a^	*38 (11)*	*13 (4)*	*<0*.*001*
	*MAM* ^b^	*95 (29)*	*35 (10)*	
	*No malnutrition*	*200 (60)*	*285 (86)*	
*WHZ* ^c^	*<−3SD*	*49 (8)*	*5 (1)*	*<0*.*001*
	*Between −3 et −2SD*	*56 (17)*	*9 (3)*	
	*>−2DS*	*228 (68)*	*319 (96)*	
*Socio-economic class*	*Lowest income*	*14 (4)*	*26 (8)*	*0*.*001*
	*Middle income*	*245 (74)*	*267 (80)*	
	*Highest income*	*74 (22)*	*40 (12)*	
*Mother completed primary school*		*222 (67)*	*170 (51)*	*<0*.*001*
*Cohabiting parents*		*25 (8)*	*10 (3)*	*0*.*004*
*Access to improve water*		*172 (52)*	*131 (39)*	*0*.*001*
*Diet*				
*< 6 months (n = 95)*		*n = 57*	*n = 38*	*0*.*055*
	*Exclusive breast feeding*	*9 (16)*	*6 (16)*	
	*Mixed feeding*	*8 (14)*	*1 (3)*	
	*Dietary diversification*	*40 (70)*	*31 (81)*	
*≥ 6 months (n = 538)*		*n = 276*	*n = 295*	
	*Exclusive breast feeding*	*1 (0*.*4)*	*2 (0*.*7)*	
	*Mixed feeding*	*0 (0)*	*0 (0)*	
	*Dietary diversification*	*275 (99*.*6)*	*293 (99*.*3)*	

^a^ Severe acute malnutrition: MUAC<115mm

^b^ Moderate acute malnutrition: 115≥MUAC≤125mm

^c^ Weight for length Z-score

The mean time since onset of diarrhea before cases were presented at the hospital was 3.7 (±1.8) days. Cases presented the following clinical symptoms: severe dehydration (N = 239, 72%); hemodynamic shock (N = 216, 65%); serious neurological injury (N = 239, 72%); fever (body temperature ≥38°) (N = 264, 79%); vomiting (N = 267, 80%); and extra digestive signs (N = 94, 28%), including 69 cases of upper respiratory tract infection, 5 cases of pulmonary signs, 8 cases of digestive signs and 12 cases of other signs. 26 cases (8%) were positive for malaria, all of them due to *Plasmodium falciparum*. The mean episodes of vomiting in the last 24 hours were 4.2 (±2), and the mean number of stools during the last 24 hours was 6.1 (±2). In 16 cases (5%) and 56 cases (17%), macroscopic blood and mucous were found in the stools, respectively.

### Comparison between cases and controls

Demographic characteristics of cases and controls are summarized in Table 2. Cases and controls were well balanced for age and sex at baseline. Cases were significantly more severely or moderately malnourished than controls, 40% versus 14%, respectively, p<0.001. Cases belonged to a higher socio-economic class, had more mothers who completed primary school and had more access to improved water than controls. No difference in diet was found between the two groups.

### Pathogens

The results are summarized in Table 3. At least one of the sought-for pathogens was identified in 78% and 40% of cases and controls, respectively, p<0.001. Mixed bacterial/viral infections were detected in 10% of cases and 4% of controls, p = 0.001. Viruses were the most prevalent pathogens, detected in 55% of cases and 15% of controls, p<0.001. Conversely, *Giardia intestinalis* was more frequent in controls (7.8%) than in cases (0.9%), p<0.001). The *ipaH* gene was detected in all 14 *Shigella*-positive culture specimens except one and in 67/651 (10%) culture-negative specimens. In all, 50/333 (15%) and 30/333 (9%) samples were considered positive for the entity *Shigella*/EIEC in cases and controls, respectively. Presence of *Shigella*/EIEC was significantly associated with blood in the stool (p<0.001). Diarrhoeagenic *E*.*coli* were found in as many cases as controls. Of the diarrhoeagenic *E*.*coli* pathotypes detected, the most frequent was EAEC (5.7% of cases and 4.5% of controls) followed by ETEC (4.8% of cases and 4.8% of controls). No significant difference between cases and controls for LT, ST, and LT/ST toxin-producing ETEC was found.

**Table 3:** Adjusted OR (aOR) and adjusted attributable fraction (aAF) for pathogens and risk of severe childhood diarrhea by age and globally.

		Cases n(%)	Controls n(%)	aOR^a^ (95% CI)	aAF^a^ (95%CI)
Infants		n = 195	n = 195		
	Rotavirus	90 (46.1)	8 (4.1)	44.8 (10.6–189.5)	45.0 (41.6–45.7)
	Astrovirus	25 (12.8)	8 (4.1)		
	Norovirus	18 (9.2)	9 (4.6)	3.2 (1.1–9.8)	6.3 (0.8–8.1)
	Adenovirus	11 (5.6)	9 (4.6)		
	*Shigella* spp/EIEC^b^	17 (8.7)	14 (7.2)		
	*Shigella* spp ^c^	2 (1.0)	1 (0.5)		
	*Salmonella enterica*	1 (0.5)	4 (2.1)		
	*Campylobacter* spp.	3 (1.5)	5 (2.6)		
	Diarrhoeagenic *E*. *coli*	24 (12.3)	17 (8.7)		
	*Entomoeba histolytica*	0 (0)^d^	1 (0.5)^e^		
	*Giardia intestinalis*	0 (0)	6 (3.1)		
	*Cryptosporidium parvum/hominis*	32 (16.4)	7 (3.6)	4.6 (1.8–11.7)	12.5 (7.1–14.6)
Toddlers		n = 103	n = 103		
	Rotavirus	43 (41.7)	2 (1.9)		
	Astrovirus	7 (6.8)	7 (6.8)		
	Norovirus	11 (10.7)	5 (4.8)	2.6 (0.8–8.5)	
	Adenovirus	6 (5.8)	6 (5.8)		
	*Shigella* spp/EIEC^b^	23 (22.3)	14 (13.6)		
	*Shigella* spp ^c^	7 (6.8)	1 (1.0)		
	*Salmonella enterica*	8 (7.7)	3 (2.9)		
	*Campylobacter* spp.	2 (1.9)	3 (2.9)		
	Diarrhoeagenic *E*. *coli*	10 (9.7)	13 (12.6)		
	*Entomoeba histolytica*	1 (1.0)^d^	3 (2.9)^e^		
	*Giardia intestinalis*	2 (1.9)	12 (11.6)	0.16 (0.03–0.7)	9.7 (3.5–11.3)
	*Cryptosporidium parvum/hominis*	7 (6.8)	2 (1.9)	2.9 (0.6–14.8)	
Children		n = 35	n = 35		
	Rotavirus	1 (2.8)	1 (2.8)		
	Astrovirus	1 (2.8)	0 (0)		
	Norovirus	4 (11.4)	0 (0)		
	Adenovirus	2 (5.7)	3 (8.6)		
	*Shigella* spp/EIEC^b^	10 (28.6)	2 (5.7)	4.1 (0.9–19.4)	
	*Shigella* spp ^c^	2 (5.7)	2 (5.7)		
	*Salmonella enterica*	4 (11.4)	1 (2.9)	2.3 (0.2–23.4)	
	*Campylobacter* spp.	0 (0)	1 (2.9)		
	Diarrhoeagenic *E*. *coli*	3 (8.6)	5 (14.3)		
	*Entomoeba histolytica*	4 (11.4)^d^	2 (5.7)^e^		
	*Giardia intestinalis*	1 (2.8)	8 (22.9)	0.17 (0.02–1.4)	
	*Cryptosporidium parvum/hominis*	3 (8.6)	0 (0)		
Total		n = 333	n = 333		
	Rotavirus	134 (40.4)	11 (3.3)	51.8 (15.6–172.6)	39.2 (37.4–39.8)
	Astrovirus	33 (9.9)	15 (4.5)	3.4 (1.05–10.8)	7.0 (0.5–8.9)
	Norovirus	33 (9.9)	13 (3.9)	3.0 (1.3–7.2)	6.6 (2.3–8.5)
	Adenovirus	19 (5.7)	18 (5.4)		
	*Shigella* spp/EIEC^b^	50 (15.0)	30 (9.0)	2.4 (1.3–4.5)	8.7 (3.5–7.7)
	*Shigella* spp ^c^	11 (3.3)	3 (0.9)	3.4 (0.7–17.6)	
	*Salmonella enterica*	13 (3.9)	8 (2.4)		
	*Campylobacter* spp.	5 (1.5)	9 (2.7)		
	Diarrhoeagenic *E*. *coli*	37 (11.1)	35 (10.5)		
	*Entomoeba histolytica*	5 (1.5)^d^	6 (1.8)^e^		
	*Giardia intestinalis*	3 (0.9)	26 (7.8)	0.2 (0.05–0.7)	6.2 (2.3–7.4)
	*Cryptosporidium parvum/hominis*	42 (12.6)	9 (2.7)	6.0 (2.4–14.6)	10.5 (7.3–11.7)

^a^ Adjusted on the presence of others pathogens

^b^ Determined by ipaH PCR

^c^ Determined by culture

^d^ Trophozoite

^e^ Cystic form

Other pathotypes (EPEC, ATEC, EIEC and STEC) were found in less than 3% of the children. *Cryptosporidium parvum/hominis* was identified more frequently in cases than in controls (12.6% of cases and 2.7% of controls, p<0.001).

### Odds-Ratio and attributable fraction

In multivariate analyses, when adjusted on the presence of other pathogens, five pathogens were positively associated with diarrhea: rotavirus, norovirus, astrovirus, *Shigella*/EIEC, *Cryptosporidium parvum/hominis* and one appeared negatively associated: *Giardia intestinalis*. The adjusted AFs (aAF) were 39% for rotavirus, 7% for both norovirus and astrovirus, 9% for *Shigella*/EIEC and 10% for *Cryptosporidium parvum/hominis*. The protective fraction for *Giardia intestinalis* was 6%, (Table 3).

### Relation between pathogens and age

**Fig 2:**
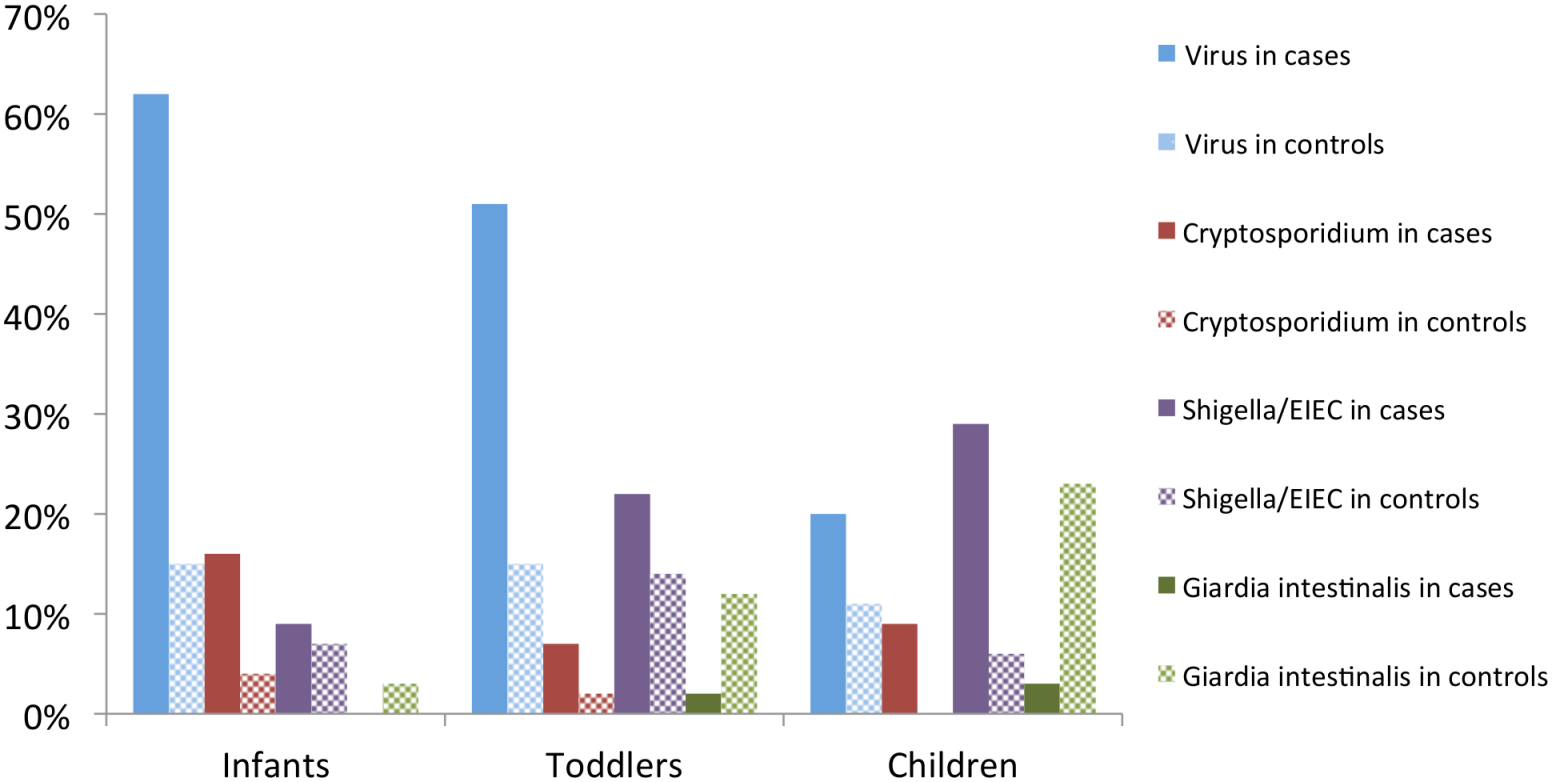
Prevalence of main pathogens by age category among cases and controls.

In cases, the prevalence of pathogens varied according to age categories (Fig 2). Globally, the prevalence of viruses and *Cryptosporidium parvum/hominis* in cases decreased with age, whereas they increased for *Shigella*/EIEC and *Giardia intestinalis*. In cases, viruses were found in 63% (122/195) of infants, 50% (52/103) of toddlers and 20% (7/35) of children; (p<0.001). *Cryptosporidium parvum/hominis* were found in 16% (32/195) of infants, 7% (7/103) of toddlers and 9% (3/35) of children, (p = 0.04). *Shigella*/EIEC were found in 9% (17/195) of infants, 22% (23/103) of toddlers and 29% (10/35) of children, (p<0.001). In controls, the percentage of virus was stable with age, and the proportion of *Cryptosporidium parvum/hominis* remained low. In contrast, the percentage of *Giardia intestinalis* was higher among controls than in cases and increased with age.

### Relation between the main pathogens and seasonality

**Fig 3:**
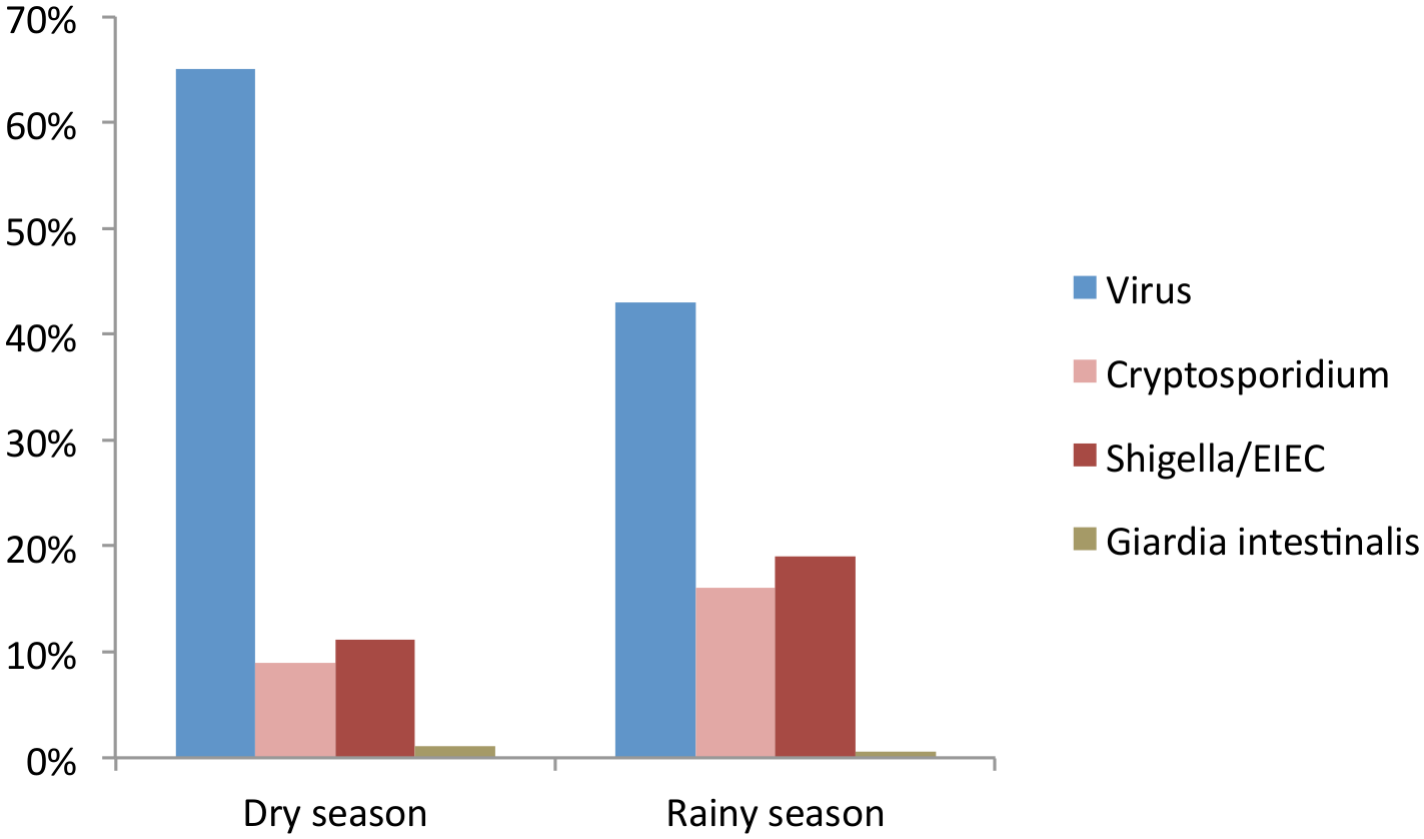
Prevalence of main pathogens by season among cases.

Viruses were more frequently identified during the dry season, with 65% (115/179) of cases compared to 43% (66/154) in rainy season, p<0.001. In contrast, *Shigella*/EIEC and *Cryptosporidium parvum/hominis* were mainly identified during the rainy season: for Shigella, 19% (30/154) cases in the rainy season and 11% (20/179) in the dry season (p = 0.03); and for *Cryptosporidium parvum/hominis*, 16% (25/154) cases in the rainy season and 9% (17/179) in the dry season (p = 0.06) (Fig 3).

### Treatments

**Fig 4:**
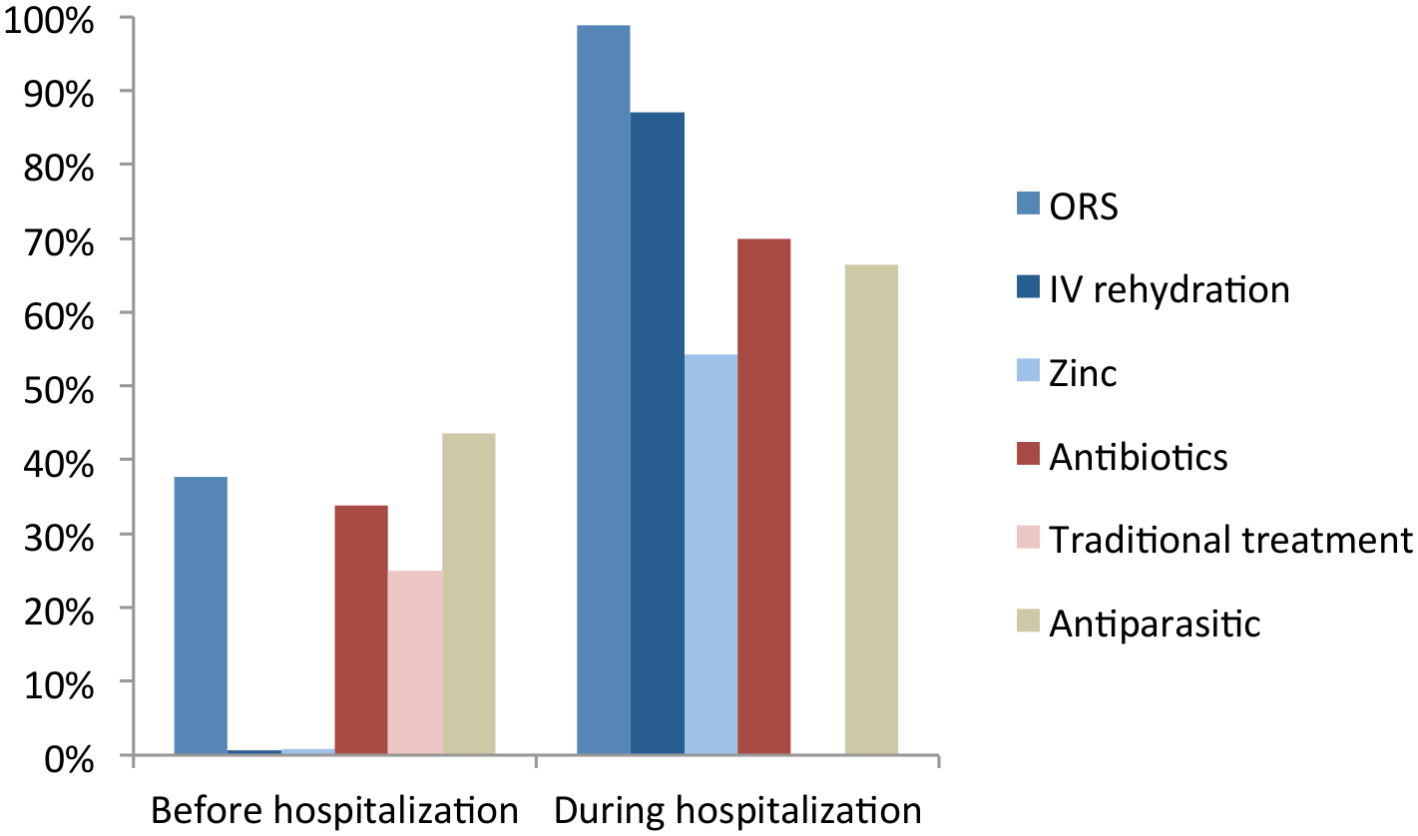
Treatments before and during hospitalization.

Before hospitalization, ORS and zinc supplementation were prescribed to 38% and 0.9% children, respectively. Antiparasitic treatments and antibiotics were administered to 44% and 34% of children, respectively. One on four children (25%) received traditional treatments that consisted mainly in infusions or herbal decoctions (72%), herbal enemas (16%) or fruit porridge (9%). During hospitalization, 99% of children received ORS, 87% intravenous rehydration, 70% antibiotics, 66% antiparasitic treatments, 55% zinc, and none received traditional treatments (Fig 4). The only factor identified as independently associated with the prescription of ORS before hospitalization was bloody stools. No factors were identified to be associated with the use of antibiotics before hospitalization or the use of any treatment during hospitalization.

### Outcome

The mean duration of hospitalization was 4.8 (±2.8) days. Four percent of children (12/333) died during hospitalization and 1% (3/271; 50 missing values) died during the two months after discharge. Among the 12 deaths, 5 were infants (< 11 months), 5 were toddlers (12 to 23 months), and 2 were children (24–59 months). Three cases were positive for *Plasmodium falciparum* and probably died of severe malaria, 2 from severe anemia, 3 from septic shock, and 1 from small bowel obstruction. Finally, 3 died from metabolic acidosis secondary to hemodynamic or septic shock. In the multivariate analysis, the only factor found to be significantly associated with the vital status was the nutritional status at the time of hospitalization: 11% (4/38) of children with SAM (WHZ < -3DS or MUAC < 115mm) died during hospitalization, compared to 3% (8/293) for MAM or normally well-nourished children, (p = 0.026).

## Discussion

This study is the first case-control study conducted in CAR that provides the etiology and clinical outcome of children of less than five years that were hospitalized for diarrhea in Bangui. A large prospective study, the GEMS study, used a similar approach with matched case-control in children between 0 to 59 months suffering from moderate-to-severe diarrhea in seven countries, including four in Africa: The Gambia and Mali (West Africa), Mozambique (South Africa) and Kenya (East Africa) [11]. Our study differs from GEMS by its recruitment. No country from Central Africa was included in the GEMS study. In addition, our study was conducted in a very low-income country, suffering from long-term instability, substantial poverty, and resource-poor primary health centers. Finally, the study also differs from the GEMS study in its inclusion criteria. In our study, children were included because they were hospitalized for diarrhea, whether or not they met the WHO criteria for severe dehydration [12]. This inclusion criterion, easier to apply in resource-poor settings, fits well with the WHO criteria for severe diarrhea. Indeed, an overwhelming majority of cases had severe dehydration (72%), more than 60% had signs of hemodynamic shock and almost 90% had to be intravenously rehydrated. In this way, our study complements the GEMS study.

HIV positive children were excluded from the present study. Indeed, HIV (co-) infection increases the risk of severe diarrhea by impairing the immune system, making problematic the interpretation of results in the context of case/control comparison. Moreover, it was not possible to test the community controls for HIV without returning to provide individual announcement and counseling, which was not possible in our study. Even if the HIV status of the controls was unknown, we supposed that it was low because only 22 hospitalized cases were tested positive for HIV and the children selected as controls were in good health. Among the 406 HIV-negative cases, only 333 were included in our analysis because they were well-matched with the controls as shown in the flow chart. The main criterion for impaired matching was age. The majority of these cases were borderline, with only few days or weeks of difference between cases and controls. The addition of the wrongly matched children for age and sex did not modify the results. In the current analysis, we prefer to maintain the criteria that were defined in the protocol.

At least one of the sought-for pathogens was identified in around 80% of children hospitalized for diarrhea, and approximately one in ten cases presented mixed bacterial/viral co-infections, consistent with findings in other African countries [13, 14]. Five pathogens were significantly associated with severe hospitalized diarrhea, namely rotavirus, norovirus, astrovirus, *Shigella*/EIEC, *Cryptosporidium hominis/parvum*. Seasonality and age effects were also observed, consistent with other studies [9, 15–17]. These results may assist clinicians diagnosing the causes of diarrhea. The frequency of *Giardia intestinalis* detection was lower in children with diarrhea (0.9%) than in asymptomatic carriers (7.8%), a finding also in keeping with previous data [18, 19] and supporting claims that this parasite is not a major cause of severe diarrhea. Further studies are needed to determine whether *Giardia intestinalis* protects against or is a consequence of diarrhea. As in GEMS [11], most attributable episodes of diarrhea were associated with rotavirus (40% of the cases versus 3.3% in controls) in concurrence with previous data from CAR [20, 21] and other Sub-Saharan countries [15]. Most rotavirus infections occurred in the youngest children, consistent with the limited protection conferred by maternal antibodies during the first months of life and the effective immunity granted by repeated infections [22]. The incidence and severity of rotavirus infections has declined significantly in countries that have integrated the rotavirus vaccine into their routine childhood immunization policies [23], highlighting the urgent need to introduce it within the CAR’s Expanded Program on Immunization [24]. The proportion of norovirus infections among cases and controls was slightly lower than the prevalence reported from high-mortality developing countries (about 14% in cases versus 7% in controls) [16].

*Cryptosporidium*, a major cause of chronic diarrhea in malnourished patients or those with positive HIV status [25], was a significant pathogen in our study. Our findings on *Cryptosporidium* are consistent with those from GEMS [11] and indicate the high global burden of cryptosporidiosis among children in Africa, regardless of their HIV-status. These elements support the need to inform healthcare professionals about this pathogen and to develop practical, inexpensive kits for its detection in resource-poor settings.

*Shigella* spp. are consistently reported as highly associated with diarrhea in case-control studies [11, 26, 27]. In our study, *Shigella* spp are the third most important pathogen to be associated with diarrhea, after rotavirus and *Cryptosporidium*. The proportion of *Shigella*/EIEC infections among cases and controls was 15% and 9%, respectively, indicating that the prevalence of asymptomatic shedders is higher than expected. However, comparing our findings with those of other studies is difficult, because the pathogen was largely detected using PCR. Traditionally, the current gold standard for *Shigella* species detection is culture, which is highly selective, but poorly sensitive due to inconsistent bacterial load, loss of bacterial viability during specimen transport and frequent antibiotic treatment before culture. In addition, the gene *ipaH* used for the diagnosis is also carried by EIEC, implying that PCR cannot differentiate between Shigellosis and EIEC. Nevertheless, it is well established that *Shigella* is much more prevalent and thus, probably represents most of the *ipaH*-associated organisms detected. Usually, PCR-based methods have a higher sensitivity compared to conventional culture methods, which improves the ability to detect pathogens in a stool sample. As individuals with diarrhea tend to have higher quantities of bacteria isolated from their stool than do those without diarrhea [28–30], the *Shigella*-specific disease burden might be underestimated using qualitative PCR. A recent study showed that a cutpoint threshold of approximately 1.4 × 10^4^ *ipaH* copies could be the new reference standard for the detection and diagnosis of shigellosis in children in low-income countries [31].

In case-control studies, diarrhoeagenic *E*. *coli* pathotypes show inconsistent association with diarrhea patients, whereas *Salmonella enterica*, *Campylobacter* and adenovirus are often found in similar proportions in patients with or without diarrhea, as we observed in our data [10, 11, 19, 26]. This finding suggests that comparing prevalence between cases and controls may have little value for pathogens with frequent asymptomatic excretion [32, 33].

As reported in previous studies [34, 35], acute malnutrition was associated with severe diarrhea and was shown to be a risk factor for death during hospitalization. This finding is of major concern, because in 2012 in CAR, 8% of the children under five years suffered from acute malnutrition and 39% of chronic malnutrition [36]. This situation has likely worsened because the country has experienced civil war since March 2013.

Our findings also shed light on the management of severe childhood diarrhea. Although the WHO recommends the use of antibiotics in children with bloody diarrhea (5% of cases in our study), suspected cholera, or associated sepsis, we found that 40% of children before their arrival and 70% during hospitalization received an antibiotic treatment. Combined with the widespread use of antiparasitic treatments before consultation (44%), this finding could partially account for the relatively low number of bacteria or parasites found among cases. However, the bias was minimized for *Shigella* species and *Cryptosporidium parvum/hominis* as they were detected using PCR.

Furthermore, the uncontrolled consumption of antimicrobial agents is cause for concern in countries like the CAR with inadequate healthcare systems, because it favors the spread of antimicrobial resistance [37–39]. Antibiotic treatments were primarily pills obtained without prescription from street vendors, who offer also diagnosis and limited diagnostic and medical services [6]. These medicines are much less expensive than in pharmacies, but there are no oversights on the safety, appropriateness or duration of such treatments [6].

Despite guidelines that recommend the use of ORS and zinc supplementation for all children, only 40% of children received ORS and less than 1% took zinc before their hospitalization. Although the pre-hospitalization ORS finding is higher than that found in other studies (ORS before hospitalization in 16% of cases according to UNICEF in CAR between 2008 and 2012, 20% in Senegal), it is essential to continue education of mothers on the importance of rehydration and zinc in home management of diarrhea.

The WHO also recommends exclusive breastfeeding for the first six months of life. Only 16% of children under six months old were exclusively breastfed, whereas others were exposed to putative pathogens in weaning foods or inadequate diversity of complementary foods.

Our study has limitations. We could not complete the planned inclusion of 600 cases and 600 controls, mainly due to security problems in Bangui. Only 2 or 3 children were recruited per day during the study-period. This low recruitment can be explained by the cost associated with transport to the hospital, hospitalization, treatment and care. As previously described [6], children afflicted with severe diarrhea have a complex therapeutic itinerary. On average, parents bring their diarrheic children to the hospital after three days of symptom, and after frequently receiving various street medicines or home remedies. These findings are confirmed by the socio-economic differences observed between cases and controls, the cases being “less poor” than the controls. It is likely that a certain number of children never go to the hospital because their parents cannot pay. Moreover, at a time where ORS was widely adopted in the community, it is not surprising that children with moderate diarrhea that predominates in Bangui were not hospitalized, and therefore were not cases included in our study. The high childhood mortality rate reported in our study (4%) may be even higher for children with poor access to healthcare services.

The choice of immunological methods to detect rotavirus, astrovirus, adenovirus and norovirus is questionable. Indeed, some more sensitive molecular methods have been used in other epidemiological studies on diarrhea [40, 41]. However, the impact of using lower sensitivity methods on the interpretation of the results is likely to be low. The sporadic low level viral shedding is not necessarily clinically relevant and if detected using highly sensitive methods can complicate the interpretation of the results. As described above for *Shigella* species, high sensitivity of PCR-based methods could lead to an underestimation of virus-specific disease burden when comparing cases with controls. Nevertheless, quantitative methods would have been very useful to assess the relationship between virus shedding and clinical severity in our study [42].

The data reported here are particularly important, given the significance of childhood diarrhea in countries with inadequate healthcare systems and long-term instability, as well as the lack of high-quality data and the difficulty of carrying out such studies in these contexts. Rotavirus, norovirus, astrovirus, *Shigella*/EIEC, *Cryptosporidium hominis/parvum* and *Giardia intestinalis* were significantly associated with severe hospitalized diarrhea. Because rotavirus was the most common cause of severe diarrhea, the introduction of the rotavirus vaccine in CAR will certainly have a major impact on childhood diarrhea. Severe diarrhea requiring antibiotics was extremely rare among CAR children under five years old. The observed overuse of antibiotics poses a major risk for the emergence of resistance, particularly when new discoveries of antibiotics are almost non-existent and the risk of therapeutic impasse real. The development of new medicines, vaccines and new rapid diagnostic tests that can be conducted bed-side should be high priorities for low-resource countries, particularly those suffering from instability and poorly-functioning health systems, as well as for global health structures. Future studies measuring the impact of antibiotic overuse on the intestinal microbiome of children will help to shed light on the complex linkages between malnutrition, diarrhea and immunity.

## Supporting Information

S1 Checklist STROBE Checklist. (DOCX)
