# Supplementary material for: Etiology and Epidemiology of Diarrhea in Hospitalized Children from Low Income Country: A Matched Case-Control Study in Central African Republic
Source: PLoS Negl Trop Dis. 2016 Jan 5;10(1):e0004283. doi: 10.1371/journal.pntd.0004283 (PMC4701495; doi:10.1371/journal.pntd.0004283)
Supplement: S1 Checklist — (DOCX) [file pntd.0004283.s001.docx]

STROBE Statement—Checklist of items that should be included in reports of ***cross-sectional studies***

|  | Item number | **Recommendation** | Page |
| --- | --- | --- | --- |
| **Title and abstract** | 1 |  | 1-2 |
| **Introduction** |  |  | 3-4 |
| Background/ Rationale | 2 |  | 3-4 |
| Objectives | 3 |  | 4 |
| **Methods** |  |  | 5-9 |
| Study design | 4 |  | 5 |
| Setting | 5 |  | 5 |
| Participants | 6 |  | 5-6 |
| Variables | 7 |  | 5-8 /Table 1 |
| Data source/measurement | 8 |  | 5-8 |
| Bias | 9 |  | 5-7 |
| Study size | 10 |  | 8 |
| Quantitative variables | 11 |  | 8-9 |
| Statistical methods | 12 |  | 8-9 |
| **Results** |  |  | 10-12 |
| Participants | 13 |  | 1. 10 2. 13 3. Flow Chart |
| Descriptive data | 14 |  | 10/Table2 |
| Outcome data | 15 |  | 10-11/Table 3 |
| Main results | 16 |  | 11/Table3 |
| Other analyses | 17 |  | 10/12/Table2/Fig2/Fig3/Fig4 |
| **Discussion** |  |  | 13-17 |
| Keys results | 18 |  | 13-17 |
| Limitations | 19 |  | 13/15-16 |
| Interpretation | 20 |  | 13-17 |
| Generalisibility | 21 |  | 13-14 |
| **Other information** |  |  |  |
| Funding | 22 |  | Total Foundation |
